# A Comparative Study on Visual Detection of *Mycobacterium tuberculosis* by Closed Tube Loop-Mediated Isothermal Amplification: Shedding Light on the Use of Eriochrome Black T

**DOI:** 10.3390/diagnostics13010155

**Published:** 2023-01-02

**Authors:** Alireza Neshani, Hosna Zare, Hamid Sadeghian, Hadi Safdari, Bamdad Riahi-Zanjani, Ehsan Aryan

**Affiliations:** 1 Antimicrobial Resistance Research Center, Mashhad University of Medical Sciences, Mashhad 91967-73113, Iran; 2 Department of Laboratory Sciences, School of Paramedical Sciences, Mashhad University of Medical Sciences, Mashhad 91779-48964, Iran; 3 Medical Toxicology Research Center, Mashhad University of Medical Sciences, Mashhad 91779-48564, Iran; 4 Department of Medical Microbiology, Ghaem University Hospital, Mashhad University of Medical Sciences, Mashhad 91766-99199, Iran

**Keywords:** LAMP, *Mycobacterium tuberculosis*, visual detection, EBT, calcein, HNB

## Abstract

Loop-mediated isothermal amplification is a promising candidate for the rapid detection of *Mycobacterium tuberculosis*. However, the high potential for carry-over contamination is the main obstacle to its routine use. Here, a closed tube LAMP was intended for the visual detection of Mtb to compare turbidimetric and two more favorable colorimetric methods using calcein and hydroxy naphthol blue (HNB). Additionally, a less studied dye (i.e., eriochrome black T (EBT)) was optimized in detail in the reaction for the first time. Mtb purified DNA and 30 clinical specimens were used to respectively determine the analytical and diagnostic sensitivities of each method. The turbidimetric method resulted in the best analytical sensitivity (100 fg DNA/reaction), diagnostic sensitivity and specificity (100%), and time-to-positivity of the test (15 min). However, this method is highly prone to subjective error in reading the results. Moreover, HNB-, calcein-, and EBT-LAMP could respectively detect 100 fg, 1 pg, and 1 pg DNA/reaction (the analytical sensitivities) in 30, 15, and 30 min, while the diagnostic sensitivity and specificity were respectively 93.3% and 100% for them all. Interestingly, EBT-LAMP showed the lowest potential for subjective error in reading the results. This report helps judiciously choose the most appropriate visual method, taking a step forward toward the field applicability of LAMP for the detection of Mtb, particularly in resource-limited settings.

## 1. Introduction

Tuberculosis (TB) has been a human disease since the beginning of the Neolithic period, as studies on bones dating back over 6000 years suggest that some prehistoric people also had TB [1,2,3]. TB is caused by the acid-fast bacillus *Mycobacterium tuberculosis*, spreading via respiratory aerosols during the active pulmonary form of the disease [4]. According to the World Health Organization’s (WHO) latest annual TB report, there has been an increase in the global TB deaths between 2019 and 2021. In 2021, nearly 10.6 million new cases of TB and 1.6 million TB-related deaths were reported worldwide [5]. Treatment of TB requires a prolonged course of multiple drugs. The prompt and effective treatment of TB is crucial for controlling and preventing the emergence of potentially lethal resistant strains of *Mycobacterium tuberculosis*. To avoid delayed appropriate treatment of TB, which may facilitate disease transmission, it is crucial to diagnose infectious TB cases promptly and effectively [6]. Despite modern medical advances, the diagnosis and treatment of TB remain a formidable challenge, rendering an ancient disease a contemporary problem [7,8].

Although conventional methods such as smear microscopy and mycobacterial culture remain the major diagnostic tests for TB in resource-limited settings and most developing countries, these tests suffer from certain limitations [9,10]. As an example, smear microscopy has low sensitivity for the detection of acid-fast bacilli (AFB) in clinical specimens (5–10 × 10^4^ AFB/mL), and mycobacterial culture is a time-consuming method, taking 3–8 weeks to conclude [11].

On the other hand, a pathogen’s genome is a diagnostic biomarker for accurately identifying the causative agent of an infectious disease, and polymerase chain reaction (PCR) is the gold standard for identifying nucleic acids. To this end, PCR-based molecular methods have been widely studied to detect *Mycobacterium tuberculosis* (Mtb) with promising results [6,12,13,14]. Despite being highly sensitive and specific, these diagnostic methods have failed to replace smear and culture methods according to a report by the U.S. Centers for Disease Control and Prevention (CDC) [15].

Loop-mediated isothermal amplification (LAMP) is an attractive alternative technique for nucleic acid amplification [16] that has a promising prospect for the diagnosis of TB and has been recommended by the WHO for this purpose [17]. LAMP has several advantages over the conventional PCR-based assays: (i) it does not require DNA denaturation to amplify the target gene; (ii) the reaction is performed at a constant temperature obviating the need for expensive equipment; (iii) it is a highly specific reaction due to the use of six primers including two chimeric ones, recognizing eight distinct regions on the target sequence; (iv) the reaction is very efficient and rapid so it can generate more than 10^9^ copies of the target gene within 15–60 min of incubation at 60–65 °C; and (v) it is less affected by the presence of inhibitor substances in clinical specimens [16,18].

Consequently, LAMP can be considered as an invaluable diagnostic method for various infectious diseases [18,19,20], demonstrating a high potential for being a point-of-care test [21,22]. However, LAMP is highly prone to carry-over contamination and the amplicons of the reaction can frequently lead to false-positive results, an issue of immense practical importance [23,24]. To this end, a closed tube LAMP assay has been proposed to address this concern via different approaches [25,26,27,28]. In fact, false-positive LAMP results can be avoided by visually reading the results with the naked eye, which is considered as a primary benefit of LAMP, which eliminates the requirement to open the reaction tube. Among the various methods for detecting LAMP products, visual endpoint evaluation of the reaction based on a color change or the presence of turbidity is preferred [22].

Fluorescence or metal ion indicator dyes such as calcein or hydroxy naphthol blue (HNB) are added to the LAMP reaction mixture in colorimetric approaches, and the test result is determined by changing the color of the reaction. Among all of the colorimetric methods studied to date, calcein and HNB are the most frequently used indicator dyes in LAMP assays [20,22,26,28]. Calcein is a fluorescence dye added along with MnCl_2_ to the LAMP pre-reaction mixture. The primary color of the reaction is orange, since the combination of calcein with Mn^2+^ ions quenches calcein fluorescence. Manganese ions are replaced by Mg^2+^ ions as the LAMP products are produced due to an increased concentration of pyrophosphate ions in the reaction, which deprives the calcein of Mn^2+^ ions. The reaction turns yellow under visible light at this point [29]. Alternatively, HNB is a metal indicator whose color is determined by the concentration of Mg^2+^. When magnesium ions are present, it turns violet; however, as the LAMP reaction proceeds, pyrophosphate ions are produced and combined with magnesium to form a magnesium pyrophosphate precipitate. The color of the reaction changes from violet to sky blue as the Mg^2+^ level decreases [26].

In the turbidimetric method, the white turbidity or precipitate of magnesium pyrophosphate as a LAMP by-product of the LAMP reaction indicates the reaction’s positivity, which can be determined visually or with an optical instrument [27]. Both colorimetric and turbidimetric methods are observable with the naked eye [26,27,28,30].

Occasionally, DNA amplification may occur due to the formation of primer dimers, producing turbidity as a non-specific positive signal. Therefore, designing valid LAMP primers is critical when the turbidity method is used [22].

Obviously, the colorimetric detection of amplification products through the naked eye could increase the popularity of a molecular diagnostic tool, making it suitable for field application. Among all colorimetric detection methods, using a metal ion indicator dye is highly sensitive, straightforward, economical, and efficient. This kind of indicator is readily available and can be incorporated into the pre-reaction mixture [22,25]. However, the transition from violet to sky blue is too subtle in the HNB-mediated LAMP reaction, allowing for a subjective error in result interpretation [31]. This is also a problem with calcein as a fluorescence dye, whose color changes from orange to yellow under visible light. Therefore, as a major disadvantage, an “experienced eye” is needed to read the LAMP results while using these two colorimetric methods.

As a result, we screened other compounds to identify an alternative indicator for possible improvements in the detection of the LAMP reaction for TB diagnosis through the naked eye. In this regard, an alternative metal indicator with the same mechanism of action as HNB was identified and evaluated to detect Mtb. Eriochrome black T (EBT) is a newer and less studied magnesium ion indicator dye that can be directly added to the LAMP mixture to interpret the reaction result visually. Only one brief report on using EBT for the visual detection of Mtb by LAMP assay exists [32]. In this prototype study, however, the practical conditions of the optimal reaction and its performance on clinical specimens compared to the most frequently employed LAMP monitoring methods were not clarified.

Considering these facts, the present study aims to determine the optimal conditions of the EBT-mediated LAMP reaction and its diagnostic performance for visually detecting Mtb through naked eye observations. This study was conducted to determine whether EBT is an appropriate alternative to HNB/calcein to be applied in LAMP. In addition, we designed a closed tube LAMP assay to eliminate carry-over contamination from previous LAMP assays. This is the first report to our knowledge that compares the diagnostic performance of EBT-, HNB-, calcein-, and turbidity-based LAMP assays for the diagnosis of TB. The results may pave the way for the field application of LAMP technology for the rapid, dependable, and cost-effective detection of Mtb, particularly in settings with limited resources.

## 2. Materials and Methods

**Figure 1:**
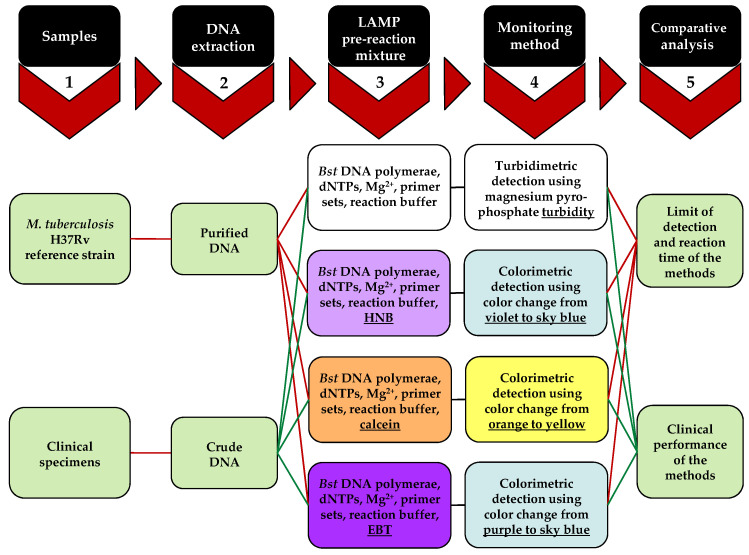
Flowchart representing the experimental approach including stages and methods utilized for comparative closed tube TB-LAMP using the four naked eye methodology for amplification detection. LAMP: loop-mediated isothermal amplification; HNB: hydroxy naphthol blue; EBT: eriochrome black T.

A flow chart representing the experimental approach of this study is shown in Figure 1. In detail, a primary step for LAMP reaction optimization is to prepare a template DNA after choosing the appropriate primer sets. Purified and crude DNA were obtained from Mtb cells and clinical specimens, respectively. LAMP primers can be newly designed using PrimerExplorer V4 software (Eiken Chemical Co. LTD, Tokyo, Japan) freely available from the Eiken Chemical Co. (https://primerexplorer.jp/e/, accessed on 21 December 2022), or obtained from previous studies, as conducted in this study [33]. Six primers were used in the LAMP reaction including two inner (FIP and BIP), two outer (F3 and B3), and two loop primers (FLP and BLP). Other necessary components of the pre-reaction mixture, in addition to the primer sets, are deoxyribonucleotides (dNTPs), Bst DNA polymerase, and a reaction buffer containing MgSO_4_, used for the turbidimetric method. Additionally, HNB, calcein, and EBT were each individually added to the pre-reaction solution of the relevant LAMP assays to be monitored via colorimetric methods. In particular, EBT was optimized in the relevant reaction by utilizing different concentrations. Furthermore, various amounts of dNTPs and magnesium were individually added to the reaction of each method to determine their optimal levels. The optimal reaction time and limit of detection were identified for each detection method, respectively, by applying different duration times and Mtb DNA concentrations to each reaction. All reactions were performed at a constant temperature of 64 °C without requiring denaturation and annealing temperatures. Finally, each monitoring method was independently applied to the clinical specimens to evaluate their clinical performance and analyzed by StatsDirect version 3 (StatsDirect Ltd., Wirral, UK) and the McNemar’s chi-square test.

### 2.1. DNA Extraction

Genomic DNA used in the present study was extracted and purified from the *M. tuberculosis* H37Rv reference strain to optimize the LAMP reactions and determine the limit of detection (LOD) of each method. For this purpose, mycobacterial colonies were suspended in TE buffer (10 mM Tris-HCl, pH 8.0, and 1 mM EDTA) and heat killed at 80 °C for one hour. The mixture was incubated overnight at 37 °C in the presence of 1 mg/mL lysozyme (Sigma-Aldrich, St. Louis, MO, USA) while shaking. Bacterial lysis was performed by the addition of 1.5% SDS and 100 µg/mL proteinase K (Fermentas Life Sciences, Vilnius, Lithuania), followed by incubation at 65 °C for 10 min. Then, the suspension was treated by 5 M NaCl and CTAB-NaCl solution (10% CTAB plus 0.7 M NaCl) at 65 °C for another 10 min. Genomic DNA was purified and precipitated, respectively, using chloroform–isoamyl alcohol (24:1) and isopropanol. Finally, the pellet was washed with 70% ethanol and dissolved in 50 µL of TE buffer for use in the subsequent experiments [34].

To determine the clinical performance of each assay, clinical specimens were initially processed by Petroff’s method [35]. Then, DNA was extracted from the clinical specimens by a simple boiling method described by Afghani and Stutman [36]. Briefly, after washing each specimen with TE buffer twice, the pellet was boiled for 5–10 min followed by a quick spin down of the tube. The supernatant was kept at −20 °C before use as the template DNA in the subsequent experiments.

### 2.2. LAMP Primers and Assays

The LAMP reactions were carried out using six primers targeting the *M. tuberculosis* 16S rRNA gene as previously described by Pandey et al. [33]. The primer sequences were as follows: F3, 5′-CTGGCTCAGGACGAACG-3′; B3, 5′-GCTCATCCCACACCGC-3′; FIP, 5′-CACCCACGTGTTACTCATGCCAGTCGAACGGAAAGGTCT-3′; BIP, 5′-TCGGGA- TAAGCCTGGACCACCAGACATGCATCCCGT-3′; FLP, 5′- GTTCGCCACTCGAGTAT- CTCCG-3′; and BLP, 5′-GAAACTGGGTCTAAATACCGG-3′.

**Figure 2:**
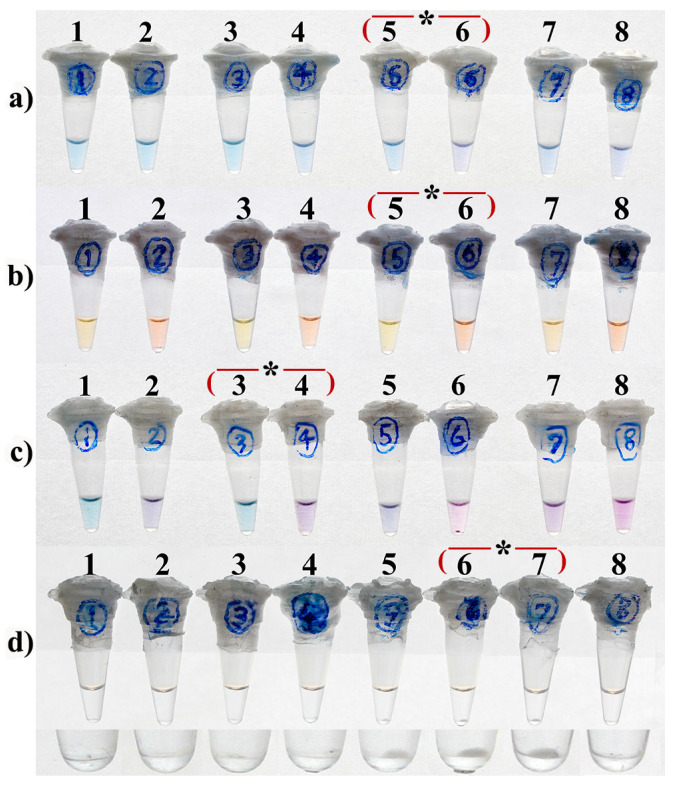
Optimization of Mg^2+^ concentration in HNB-LAMP (**a**), Calcein-LAMP (**b**), EBT-LAMP (**c**), and turbidity-LAMP (**d**) for the detection of *M. tuberculosis*. (**a**,**c**) 2.5 mM (tubes 1 and 2), 3.5 mM (tubes 3 and 4), 4.5 mM (tubes 5 and 6), and 5.5 mM (tubes 7 and 8) MgSO_4_. (**b**) 4 mM (tubes 1 and 2), 6 mM (tubes 3 and 4), 8 mM (tubes 5 and 6), and 10 mM (tubes 7 and 8) MgSO_4_. Tubes with even and odd numbers, respectively, represent negative and positive reactions. In (**d**), tubes 1 to 7 are positive reactions, respectively, containing 2, 3, 4, 5, 6, 8, and 10 mM MgSO_4_, and tube 8 is the negative control. The image on the bottom shows the close-up view of (**d**) focused on the pellets of pyrophosphate magnesium. The asterisks indicate the optimal concentration of Mg^2+^.

**Figure 3:**
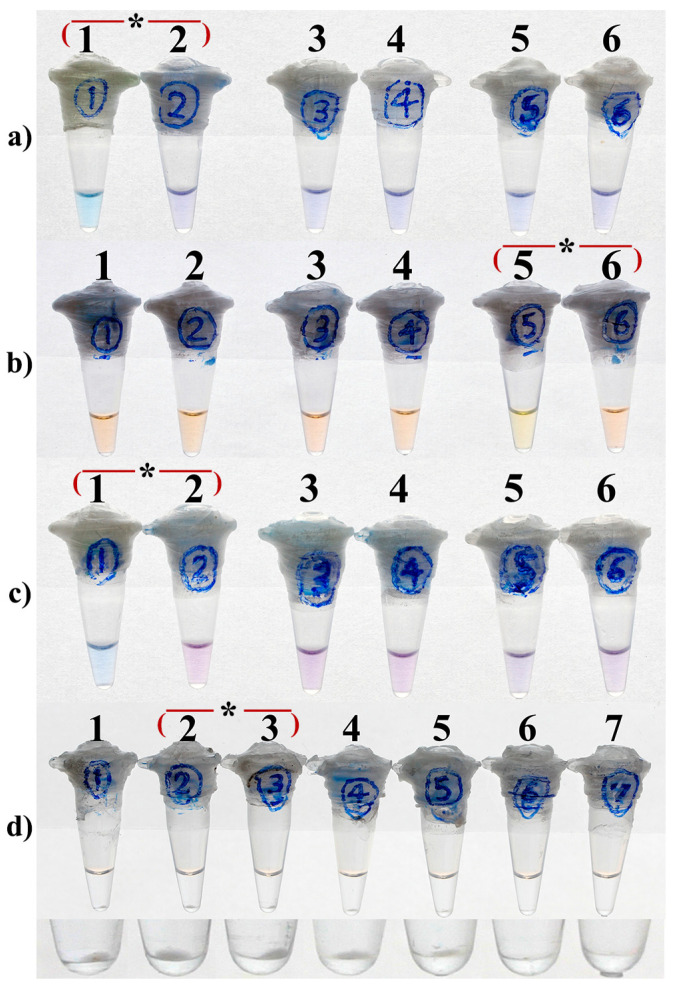
Optimization of the dNTP concentration in HNB-LAMP (**a**), Calcein-LAMP (**b**), EBT-LAMP (**c**), and turbidity-LAMP (**d**) for the detection of *M. tuberculosis*. (**a**–**c**) 0.5 mM (tubes 1 and 2), 1 mM (tubes 3 and 4), and 1.4 mM (tubes 5 and 6) dNTPs. Tubes with even and odd numbers, respectively, represent negative and positive reactions. In (**d**), tubes 1 to 6 are positive reactions, respectively, containing 0.5, 1, 1.4, 2, 2.5, and 3 mM dNTPs, and tube 7 is the negative control. The image on the bottom shows the close-up view of (**d**) focused on the pellets of pyrophosphate magnesium. The asterisks indicate the optimal concentration of Mg^2+^.

LAMP assays were performed in a total volume of 25 µL containing 1.6 µM each of the inner primers (FIP and BIP), 0.2 µM each of the outer primers (F3 and B3), 0.8 µM each of the loop primers (FLP and BLP), 0.8 M betaine (Sigma-Aldrich, St. Louis, MO, USA), 1X ThermoPol reaction buffer (New England Biolabs, Ipswich, MA, USA), 8 U *Bst* DNA polymerase (New England Biolabs), and 1 ng purified DNA from *M. tuberculosis* H37Rv. To determine the optimal condition leading to the most distinct visual result, various concentrations of MgSO_4_ (Figure 2) and dNTPs (Figure 3) were also applied to the LAMP reactions of each monitoring method.

For colorimetric LAMP reactions, 25 µM calcein (Sigma-Aldrich) plus 0.5 mM MnCl_2_ (Sigma-Aldrich), and 9 mM HNB (Sigma-Aldrich) were also added to calcein- and HNB-LAMP assays, respectively.

Due to the limited studies, the EBT concentration was additionally optimized in the relevant reaction (Table 1). For this purpose, 40, 60, 80, 100, and 120 mM EBT (Sigma-Aldrich) were individually applied to the LAMP reaction along with a negative control for each concentration. The negative controls contained all components of the reaction except the template DNA, which was replaced by sterile distilled water. The optimal concentration of EBT was determined by visually inspecting the reaction tubes every 15 min up to 180 min to obtain the most distinct color change between the test tube and its relevant negative control.

The color changes in the reactions from orange to yellow in calcein-LAMP, violet to sky blue in HNB-LAMP, and purple to sky blue in EBT-LAMP were considered as a positive result.

For turbidity-LAMP, the presence and the size of a white pellet at the bottom of the reaction tube following a quick spin-down were respectively considered as a positive and an optimal result (Figure 2d and Figure 3d).

All the reactions were incubated at 64 °C and the results were monitored by the naked eye. To prevent accidental opening of the reaction tubes, the tubes’ caps were kept in a sealed position by the use of Parafilm^®^ (Bemis Inc., Neenah, WI, USA). To evaluate reproducibility of the results, each assay was performed three times.

### 2.3. Limit of Detection and Optimal Reaction Time

First, the concentration of a solution containing purified DNA from *M. tuberculosis* H37Rv was measured three times at 260 nm by a NanoDrop ND-1000 Spectrophotometer (NanoDrop Technologies Inc., Rockland, DE, USA). Afterward, the average of these values was considered as the true concentration to prepare a 10-fold serial dilution of Mtb DNA in 10 mM Tris-HCl (pH 8.8), ranging from 100 ng/µL to 1 fg/µL. To determine the limit of detection (LOD) of the LAMP assays, 1 µL of each dilution was applied to the optimal reactions of each monitoring method as the template DNA. For all of the monitoring methods, the LOD was determined in a single day using the same serial dilution to ensure comparability of the results. To precisely determine the LOD of the LAMP monitoring methods, 20 replicates of the minimum detectable concentration of Mtb DNA were tested by each method, and this concentration was confirmed as the LOD for the respective method, if positive results were obtained in ≥95% of all 20 replicates.

To determine the optimal reaction time, the results were visually inspected and recorded every 15 min for each method. The experiment was performed on two different days using two distinct set of serial dilutions to assess the intra-day reproducibility of the results.

### 2.4. Clinical Evaluation of LAMP Assays

To evaluate the clinical performance for each monitoring method of LAMP, 30 clinical specimens including 17 sputum and 13 bronchoalveolar lavage (BAL) samples were collected from TB-confirmed patients referred to the Laboratory of Tuberculosis, Ghaem University Hospital, Mashhad, Iran. All of the specimens were smear- and culture-positive for AFB and Mtb, respectively. Mycobacterial culture was used as the gold standard for laboratory confirmation of TB cases. Clinical specimens were included in our study after completion of their routine requested tests and subjected to discard.

Five microliters of crude DNA from each clinical specimen was individually applied to the optimal reaction of each monitoring method of LAMP. Then, the results of the four monitoring methods were compared. Negative and positive controls were always included in each run of the experiment. The negative control contained all reactants minus the target DNA and the positive control contained *M. tuberculosis* H37Rv purified DNA in place of the clinical specimens’ DNA.

## 3. Results

### 3.1. Optimal Concentration

As can be deduced from Table 1, both LAMP reactions containing 40 and 60 µM EBT were positive after 30 min and remained unchanged by extending the reaction time. In the presence of higher concentrations of EBT, however, LAMP was either positive after 150 min (80 µM EBT) or totally negative (100 and 120 µM EBT), probably due to the inhibitory effect of the indicator dye (Table 1). Because the presence of 60 µM EBT in the LAMP reaction yielded the most distinct color change from purple to sky blue between the negative and positive assays, it was considered as the optimal concentration of EBT in subsequent LAMP assays.

### 3.2. Optimal Concentrations of Mg^2+^

As demonstrated in Figure 2a,c, 4.5 and 3.5 mM MgSO_4_ yielded the most distinctive visual results, respectively, for the HNB-, and EBT-LAMP assays. In the same way, the optimal concentration of MgSO_4_ for the calcein-LAMP assay was determined as 8 mM (Figure 2b), while both 6 mM and 8 mM MgSO_4_ resulted in the same pellet size of magnesium pyrophosphate at the bottom of the reaction tubes for the turbidity-LAMP assay (Figure 2d, tubes 6 and 7).

### 3.3. Optimal Concentrations of dNTPs

As shown in Figure 3a–c, 0.5 and 1.4 mM dNTPs were yielded the most distinct color change between positive and negative reactions respectively for HNB-/EBT- and calcein-LAMP assays. Additionally, the optimal concentration of dNTPs for turbidity-LAMP was 1 or 1.4 mM, as both concentrations resulted in the largest size of magnesium pyrophosphate pellet at the bottom of the reaction tubes (Figure 3d, tubes 2 & 3).

### 3.4. LOD and Optimal Time of the Reactions

**Figure 4:**
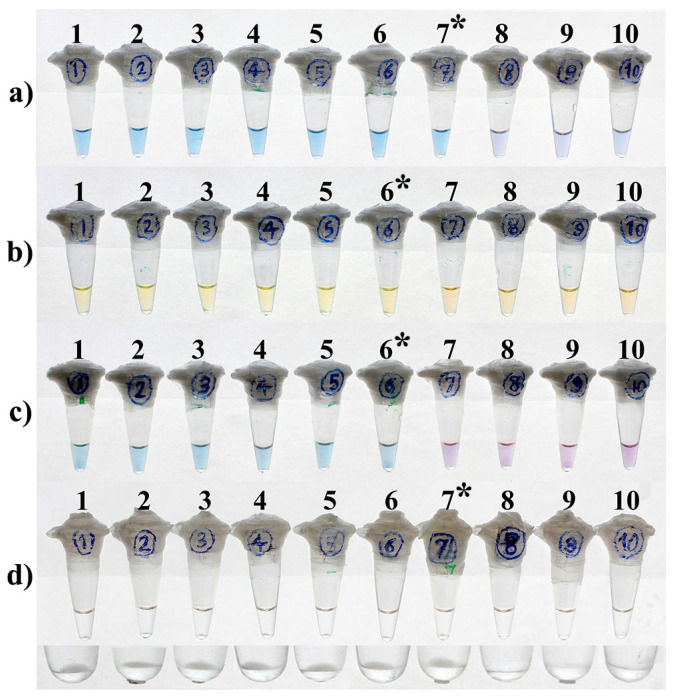
Comparison of the LOD of HNB-LAMP (**a**), Calcein-LAMP (**b**), EBT-LAMP (**c**), and turbidity-LAMP (**d**) for the diagnosis of TB. Tubes 1 to 10, respectively, contain 100 ng, 10 ng, 1 ng, 100 pg, 10 pg, 1 pg, 100 fg, 10 fg, 1 fg, and zero amount of Mtb DNA/reaction. The lower part of the figure shows the close-up view of (**d**) focused on the bottom of the tubes. The asterisks indicate the LOD of each method.

HNB- and turbidity-LAMP assays were able to detect up to 100 fg purified DNA of *M. tuberculosis* H37Rv per reaction (Figure 4a,d). This was achieved through an optimal reaction time of 15 and 30 min, respectively, for the turbidity and HNB-LAMP assays and remained unchanged by extending the duration time of the reactions (Table 2). Moreover, the LOD was 1 pg DNA/reaction for the calcein- and EBT-LAMP assays (Figure 4b,c). The optimal time to positivity of the reactions for the detection of this amount of DNA was 15 and 30 min, respectively, for the calcein- and EBT-LAMP assays. Additionally, no change was observed with extra reaction time (Table 2). In fact, calcein and EBT led to a one-log reduction in the LOD of the relevant LAMP assays compared to the turbidity- and HNB-LAMP assays.

In contrast to the calcein- and turbidity LAMP assays, no positive signal was achieved for HNB- and EBT-LAMP within the first 15 min of the amplification process, even at the higher concentrations of DNA (Table 2). By performing the experiment on two different days and on separate sets of serial dilutions of Mtb DNA, the results were shown to be reproducible. Moreover, all 20 replicates containing 100 fg/µL and 1 pg/µL Mtb DNA (the lowest detectable concentrations) were individually positive with turbidity-/HNB-LAMP and calcein-/EBT-LAMP, respectively.

### 3.5. Clinical Evaluation

In the present study, all the clinical specimens from TB-confirmed patients were positive by the turbidity-LAMP assay, with a diagnostic sensitivity of 100% (95% CI, 78.2–100%) for this method and perfect agreement between the turbidity-LAMP and mycobacterial culture as the gold standard for TB diagnosis. Only two out of thirty TB-confirmed sputum specimens were negative by the HNB-, calcein-, and EBT-LAMP. Interestingly, these two negative samples belonged to two particular TB patients whose disease could not be detected by all these colorimetric methods. Therefore, diagnostic sensitivity for the HNB-, calcein-, and EBT-LAMP assays was 93.3% (95% CI, 77.9–99.2%). Moreover, all negative controls applied to each run of the experiments were negative, indicating no false-positive result for all the LAMP detection methods evaluated in this study (specificity 100%). Finally, positive and negative predictive values were 100% and 88.2%, respectively, for the colorimetric LAMP methods.

According to the comparative analysis, the HNB-, calcein-, and EBT-LAMP assays each showed an agreement of 95.6% with a Cohen’s kappa of 0.91 with turbidity-LAMP, indicating almost complete similarity of the four monitoring methods. However, more distinct color change was observed between the positive and negative EBT-LAMP reactions, proving the lowest error due to subjectivity for this method.

## 4. Discussion

Although LAMP is superior to conventional nucleic acid amplification techniques in terms of speed and cost, it is more susceptible to carry-over contamination with secondary LAMP products, preventing widespread application [23,24,25]. This is due to the high efficiency of the reaction, which produces about 100–1000 times more amplicons than other methods such as PCR [37].

Observing the ladder-like pattern of the amplicons during agarose gel electrophoresis of LAMP products stained with a DNA intercalating dye can be used as a reference method for evaluating the assay result [16]. However, it cannot be used as the monitoring method of choice for LAMP because of the need to open the reaction tube cap and subsequent laboratory contamination with LAMP products. Frequently, the contamination problem is so severe that replacing micropipettes, pipette tips, reagents, and tubes and even relocating the testing area is essential [24]. Obviously, this will add some costs to an intrinsically inexpensive technique.

Therefore, improving LAMP monitoring methods is a research priority to facilitate the field application of this invaluable technology. However, care must be taken not to sacrifice LAMP’s advantageous characteristics such as its simplicity and cost-effectiveness.

In order to introduce a reliable monitoring method for LAMP, we employed a closed tube system to prevent the contamination of subsequent experiments with amplicons. Consequently, gel electrophoresis analysis was not the method of choice to monitor LAMP products in the current study.

To date, several strategies have been proposed to address the contamination problem utilizing a closed tube system such as the LAMP monitoring method [25,26,28,38]. This system does not allow for the opening of the reaction tube cap for the reasons above-mentioned. Instead, the result of the LAMP assay was determined through the inspection of turbidity or the color change of the reaction. This approach will certainly prevent obtaining false-positive results in downstream LAMP assays [25,38].

Mori et al. introduced this method to improve LAMP-based assays practically for the first time. They interpreted the LAMP results using the turbidity caused by the formation of magnesium pyrophosphate in the reaction. According to reports, this turbidimetric method can be used with the naked eye or, for greater accuracy, a real-time turbidimeter device. Additionally, Mori et al. suggested centrifuging the LAMP tubes at 6000 rpm for several seconds at the end of the reaction [27]. This would aid in a more straightforward visual judgment of the LAMP results by inspecting the white pellet of the magnesium pyrophosphate precipitate at the bottom of the reaction tube. In the current study, this method was also used for the turbidimetric LAMP method. As reported by others [19,39], we concluded that the interpretation of LAMP results based solely on the visual monitoring of the reaction turbidity is a subjective and error-prone judgment, especially while dealing with weak-positive results. We examined the white pellet at the bottom of an illuminated reaction tube with a loupe magnifier to improve this monitoring method. Our findings revealed that the visual monitoring of the reaction turbidity by this modified approach was one of the most sensitive monitoring methods of LAMP evaluated in the present study. Accordingly, this approach found the LOD of the LAMP assay to be 100 fg of the purified Mtb DNA/reaction.

Global efforts to develop a reliable method for monitoring the production of LAMP amplicons in a closed system resulted in an innovation in LAMP technology by employing calcein (plus MnCl_2_) in the reaction mixture. This metal ion indicator was initially reported in the LAMP reaction by Boehme et al. [29], and Tomita et al. [28] who published a detailed protocol. In this method, calcein is deprived of Mn^2+^ by pyrophosphate ions accumulated in the reaction during the amplification of target DNA, and instead, it binds to magnesium ions. This will result in a more intense bright green fluorescent emission of the reaction under UV light or a visible color change from orange to yellow of the reaction under visible light [28].

Further studies have shown that adding calcein and MnCl_2_ in the LAMP reaction would reduce the test’s sensitivity [40,41]. Yang et al. determined the sensitivity of the LAMP assay targeting IS*1081* for the diagnosis of tuberculous pleurisy [41]. The sensitivity was determined to be 100 fg and 1 pg of purified Mtb DNA using gel electrophoresis and the calcein visual inspection method, respectively. They achieved these results by applying both 60 and 90 min incubation times to the reactions [41]. In our study where the visual turbidimetric method was used instead of gel electrophoresis analysis, calcein-LAMP showed a 10-fold reduction in sensitivity for the detection of Mtb compared to the turbidimetric method (Figure 4b,d). This result is consistent with the findings of Yang et al. and other researchers [40,41], as we previously reported equal detection sensitivity for the turbidimetric and gel electrophoresis methods [18,24]. Two reasons have been proposed for the reduced sensitivity of the calcein-LAMP reaction: (i) the inhibition of LAMP reaction by Mn^2+^ [26,40]; and (ii) the direct interaction between calcein and dsDNA [42]. Nevertheless, calcein is widely used in various LAMP assays [37,41,43], even in the only WHO-recommended LAMP for TB (Loopamp^TM^ MTBC Detection Kit, Eiken Chemical Co., Ltd., Tokyo, Japan) [17].

HNB is an additional metal ion indicator dye initially described by Goto et al. for visually detecting lambda phage DNA using the LAMP technique [26]. This indicator turns violet in the presence of Mg^2+^ when added to the LAMP reaction. During the amplification process, a significant amount of magnesium is used to produce insoluble magnesium pyrophosphate as the main by-product of the reaction. As a result of the decrease in magnesium concentration, the reaction color transforms from violet to sky blue, signifying a positive test result.

Our findings demonstrated that HNB-LAMP was ten times more sensitive than calcein-LAMP for Mtb detection (Figure 4a,b). This is consistent with the lambda phage DNA results reported by Goto et al. [26].

In recent years, EBT’s usefulness in the LAMP process for visualizing the reaction’s result has garnered increased attention [32,44,45]. The mechanism of action for this metal ion indicator dye is comparable to that of HNB; however, EBT produces distinctive color changes between negative and positive reactions. Briefly, the addition of EBT causes the reaction solution to turn purple because it binds to magnesium ions. As the target DNA is amplified by LAMP, magnesium ions are depleted from EBT due to the production of magnesium pyrophosphate and a decrease in the level of magnesium ions. This leads to the color change of the reaction from purple to sky blue, indicating a positive LAMP assay.

Wang utilized the same set of LAMP primers as Yang et al. in the sole brief report on the use of EBT for the visual detection of Mtb via the LAMP assay [32,41]. Surprisingly, Wang reported a sensitivity of 8 fg Mtb genomic DNA for the EBT-LAMP assay using a 45-min reaction [32]. However, the study lacked an explanation for why a LAMP reaction containing the same set of primers achieved 100-fold higher sensitivity than that reported by Yang et al. More interestingly, this result was obtained with a shorter EBT-LAMP reaction time [32,41]. This is inconsistent with our findings, in which EBT-LAMP showed a 10-fold reduction in sensitivity compared to the turbidimetric LAMP assay. Additionally, we demonstrated that the shorter the reaction time, the lower the test sensitivity (Table 2). In addition, we utilized the identical set of LAMP primers as Pandey et al. In contrast to Wang’s findings, the sensitivity of turbidimetric- and HNB-LAMP in our study was comparable to that of real-time turbidimetric-LAMP (100 fg purified Mtb DNA/reaction), as determined by Pandey et al. [33].

Although EBT-LAMP was 10-fold less sensitive than turbidimetric- and HNB-LAMP in the present study, its analytical sensitivity was equivalent to that of the widely used calcein-LAMP method. In addition, reading the results of the EBT-LAMP assay was less susceptible to subjective error than the calcein- and HNB-LAMP assays (Table 3) due to the more distinct color change between its negative and positive reactions (Figure 4). This is of the utmost importance when LAMP is performed by an inexperienced individual, given that one of the primary goals of health organizations is to simplify diagnostic tests, so that they can be utilized in remote and resource-poor settings [46]. Although color change between negative and positive reactions of calcein-LAMP is more distinctive under UV light than visible light, it should be noted that the need for a UV transilluminator device would add an extra cost to the test.

Moreover, our results showed that calcein-LAMP could provide a positive result 15 min earlier than the HNB- and EBT-LAMP assays at the lowest detectable concentration of Mtb DNA (Table 2). We hypothesized that any delay in the positivity of the colorimetric LAMP assays could be attributed to the concentration of each indicator dye in the related reaction. Since the lowest concentration of indicator dye (25 µM) was applied to the calcein-LAMP assay with a time-to-positivity similar to turbidity-LAMP where no indicator dye was used (Table 3), we believe that the 10-fold lower sensitivity of the calcein- and EBT-LAMP assays in comparison to the HNB-LAMP assay may have distinct causes.

First, as previously stated, the functional calcein-LAMP assay depends on the reaction’s simultaneous use of MnCl_2_ and calcein. Thus, based on our findings, the 10-fold less sensitivity of calcein-LAMP appears more likely to be due to the inhibition of the reaction by Mn^2+^ ions than to the calcein itself since (in contrast to HNB and EBT) calcein did not cause any delay in the time-to-positivity of the LAMP reaction at the optimal concentration used in this study (Table 2). This is consistent with the explanations provided by Goto et al. [26] and Wastling et al. [40], in contrast to the earlier-mentioned study by Zhang et al. [42].

Second, EBT and HNB have similar chemical structures except that they contain one and three sulfur trioxide groups (SO_3_), respectively. In other words, the negative charge of HNB is twice that of EBT. This means that the possible interaction between EBT and the negatively charged backbone of DNA is more likely to occur in the LAMP reaction than between HNB and DNA. This appears to be the possible reason for our research’s 10-fold less sensitivity of EBT-LAMP compared to HNB-LAMP.

Superior to Wang’s study, a comparative evaluation of four monitoring methods of LAMP on the clinical specimens was also performed in the current research [32]. We showed that the clinical performance of turbidity-LAMP, lacking any indicator dye in the reaction, was perfect in the TB-confirmed cases (100% positivity rate). However, the LAMP positivity rate was slightly lower (93.3%, 28/30) for all of the colorimetric methods. In fact, two clinical specimens produced false-negative results for all of the colorimetric methods in contrast to the turbidimetric LAMP. We hypothesized that these two samples contained inhibitory substances that interacted synergistically with the indicator dyes or Mn^2+^ ions, in the case of calcein-LAMP, to inhibit the colorimetric reactions. Since these two false-negative results were associated with two specific clinical specimens, our opinion is more probable. Practically, the sensitivity of colorimetric LAMP could be enhanced by adding substances such as guanidine chloride to the reaction, as reported elsewhere [31]. Finally, it should be noted that the clinical performance of a diagnostic tool might be lower for extrapulmonary TB specimens than the pulmonary specimens used in our study. This is due to the paucibacillary nature of the extrapulmonary TB specimens [13]. Therefore, future study needs to be performed to reveal the diagnostic performance of the mentioned LAMP monitoring methods for the extrapulmonary TB specimens.

## 5. Conclusions

Overall, among the four LAMP monitoring methods evaluated in this study, the following conclusions can be drawn. (1) The visual turbidimetric method provided the best analytical (100 fg) and diagnostic (100%) sensitivities as well as the quickest time-to-positivity (15 min). However, it is highly prone to subjective error while interpreting the results. (2) The HNB method resulted in the highest analytical sensitivity (100 fg) among the visual colorimetric methods, although its diagnostic sensitivity (93.3%) was identical to those of calcein- and EBT-LAMP. (3) Similar to the turbidimetric method, calcein-LAMP demonstrated the shortest time-to-positivity (15 min) compared to the other two colorimetric methods (30 min). (4) The EBT method showed the lowest potential for subjective error while interpreting the results by generating the most distinct color change between negative and positive reactions under visible light. (5) EBT and HNB colorimetric methods for LAMP were performed at the lowest and highest costs, respectively (Table 3). Finally, the findings of this study may contribute to the field applicability of LAMP technology. Nonetheless, it is possible to find other chemical compounds with comparable properties such as murexide, which will need to be investigated in future studies.

## Figures and Tables

**Table 1:** Determination of the optimal concentration of EBT in the LAMP assay by applying different duration times to the reactions.

LAMP Reaction Time (min)	EBT Concentrations (µM)				
	40	60	80	100	120
15	−	−	−	−	−
30	+	+	−	−	−
45	+	+	−	−	−
60	+	+	−	−	−
75	+	+	−	−	−
90	+	+	−	−	−
105	+	+	−	−	−
120	+	+	−	−	−
135	+	+	−	−	−
150	+	+	+	−	−
180	+	+	+	−	−

Note. The most distinct color change was observed at 60 µM EBT between the positive and negative reactions after 30 min (pink-colored column). Although the reactions were positive at 40 and 80 µM EBT, respectively, after 30 and 150 min, the color change between the positive and negative reactions were less distinctive at these concentrations (grey-colored column). +, positive LAMP reaction; −, negative LAMP reaction; EBT, eriochrome black T.

**Table 2:** LAMP monitoring methods performed on various DNA concentrations/reaction with different duration times of reaction for the determination of the optimal reaction time capable of detecting the lowest amount of the Mtb DNA/reaction.

Reaction Time ^a^	Method	Mtb Purified DNA/Reaction (25 µL)									
		100 ng	10 ng	1 ng	100 pg	10 pg	1 pg	100 fg	10 fg	1 fg	0
15 min	HNB-LAMP	−	−	−	−	−	−	−	−	−	−
	Calcein-LAMP	+	+	+	+	+	+	−	−	−	−
	EBT-LAMP	−	−	−	−	−	−	−	−	−	−
	Turbidity-LAMP	+	+	+	+	+	+	+	−	−	−
30 min	HNB-LAMP	+	+	+	+	+	+	+	−	−	−
	Calcein-LAMP	+	+	+	+	+	+	−	−	−	−
	EBT-LAMP	+	+	+	+	+	+	−	−	−	−
	Turbidity-LAMP	+	+	+	+	+	+	+	−	−	−
45 min	HNB-LAMP	+	+	+	+	+	+	+	−	−	−
	Calcein-LAMP	+	+	+	+	+	+	−	−	−	−
	EBT-LAMP	+	+	+	+	+	+	−	−	−	−
	Turbidity-LAMP	+	+	+	+	+	+	+	−	−	−

+, positive LAMP reaction; −, negative LAMP reaction; Mtb, *Mycobacterium tuberculosis*. ^a^ By extending the reaction time to 120 min, no change was achieved in the results of any monitoring methods of LAMP compared to those obtained in the thirtieth minute of the reactions.

**Table 3:** A brief comparison among the four monitoring methods of LAMP used in this study for Mtb detection.

	LAMP Monitoring Methods ^a^			
	T-LAMP	C-LAMP	H-LAMP	E-LAMP
Principle	Turbidimetry	Colorimetry	Colorimetry	Colorimetry
Mechanism	Mg_2_PPi precipitation results in the formation of white turbidity in the reaction	Sequestering of Mn^2+^ from calcein results in the color change of this metal ion indicator dye	Sequestering of Mg^2+^ from hydroxynaphtol Blue leads to a color change of the indicator	Sequestering of Mg^2+^ from eriochrome black T leads to a color change of the indicator
LAMP results (negative/positive)	Clear/Turbid	Orange/Yellow	Violet/Sky blue	Purple/Sky blue
Visual inspection of results	Yes	Yes	Yes	Yes
Performing in closed system	Yes	Yes	Yes	Yes
Potential for subjective error in reading the result	High	High	High	Low
LOD ^b^	100 fg	1 pg	100 fg	1 pg
Diagnostic sensitivity (%)	100	93.3	93.3	93.3
Additional cost ^c^	None	++	++++	+
O.C. of each indicator/reaction ^d^	-	25 µM	4.5 mM	60 µM
Time-to-positivity of LAMP (min.)	15	15	30	30
Inhibitory effect on LAMP reaction	None	Yes, a 10-fold reduction in LOD at optimal concentration	No inhibitory effect at optimal concentration	Yes, a 10-fold reduction in LOD at optimal concentration

^a^ T; turbidity, C; calcein, H; HNB (hydroxynaphtol blue), E; EBT (eriochrome black T). ^b^ LOD; limit of detection. ^c^ The colorimetric LAMP reactions are slightly more costly to perform compared to the T-LAMP assay because of the additional usage of a metal ion indicator dye in each reaction. Although the extra cost was much less than a dollar, the amount varied among different colorimetric methods. ^d^ O.C., optimal concentration.

## Data Availability

Not applicable.
